# Hospitals and Medical Schools

**Published:** 1905-02-25

**Authors:** Edw. Fry, C. G. Stepney, J. Danvers Power

**Affiliations:** Hon. Secretary


### HOSPITALS AND MEDICAL SCHOOLS.

We have received a copy of the report of the Committee appointed to inquire into the financial relations between hospitals and medical schools. The Committee consisted of Sir Edward Fry, the Bishop of Stepney, and Lord Welby, and their report, addressed to the Prince of Wales, is as follows :—

Sir,—1. In October last your Royal Highness, as President of King Edward's Hospital Fund, was pleased to request us to undertake the inquiries indicated in the following Terms of Reference and Instruction :—

"To consider and report—

"1. Whether any, and if any how much, money given or subscribed for the relief of the sick poor to the 12 London hospitals having medical schools is contributed, directly or indirectly, by those hospitals, or any of them, for the maintenance of medical education.

"2. Whether any direct or indirect return for such contributions (if any) is received by the hospitals from their medical schools, and, if so, whether such return is equivalent to the amount of the contributions.

"3. Whether, in the event of the Committee finding that any hospital contributes to its medical school a sum in excess of the return it receives from the medical school, there are any special considerations advanced in justification of such expenditure, or any general considerations which would apply to all hospitals having medical schools.

"It is an instruction to the Committee to deal with the subject on the basis of the existing arrangements, and to accept from the hospitals as existing arrangements any such as they may advise the Committee will be in operation on January 1st, 1905."

2. We have the honour respectfully to submit the following report of our proceedings and of the result of our inquiries under the foregoing reference.

3. At our first meeting, on November 4th, 1904, we settled a form of return, which we addressed to the 12 London hospitals which have schools in connection with them. A copy of this form will be found in the First Appendix to this our Report.

4 On the 7th, 8th, and 10th days of December, 1904, and on the 17th day of January, 1905, we heard the oral statements of witnesses, a shorthand note of whose evidence appears in the Second Appendix to this our Report. Furthermore, we have also met on December 9th, 1904, and received and considered the replies of the 12 hospitals to the forms of return, a statement laid before us by the Hon. Stephen Coleridge, and a summary which has been prepared of the reports of the ordinary expenditure of the 16 largest London general hospitals for the year 1903.

5 The replies of the hospitals to the forms of return have undergone a close examination, and have been the subject of interviews between certain of the authorities of the hospitals and the Committee, and of correspondence between our Honorary Secretary and some of these authorities, and the original replies have in several cases been modified. The best results of this examination which we are able to supply will be found in the Third Appendix to this our Report.

6. We find it impossible to answer the first head of inquiry in the terms in which it is addressed to us for the following reasons:—That in the case of several of the hospitals (as St. Mary's) the hospital and school were founded together, and the school as well as the hospital appears to have been an object of bounty in the minds of the founders; that in some cases (as University College Hospital) the hospital was founded as an aid to the school; that in some cases (as Charing Cross and Westminster Hospitals) the school buildings were provided, or partly provided, by money subscribed for the specific purpose, or for a joint purpose of which the provision of school buildings formed a part; that in other cases sums have been contributed to hospitals, subsequent to the foundation, of which, to the knowledge of the donors, a portion was applied to the school, as *e.g.* in the case of the London Hospital where a contribution to the school has been made yearly under resolutions passed at meetings of the subscribers entitled to vote under the rules; and that, where the hospital and school form a single conjoint institution (as in the case of the Middlesex Hospital) and contributions have been made to that institution, it is obviously impossible to say how much was given for the relief of the sick poor and how much for the promotion of medical education.

7. In our investigation of the matters arising under the first inquiry we have, therefore, but of course merely for purposes of convenience, treated the question as if modified by the deletion of the words "for the relief of the sick poor," and as if all the moneys contributed to the hospitals were properly applicable to the hospital only, and not to any extent to the school.

8. But, even after eliminating this difficulty, we have found that the very intimate and complex relations which have grown up between the hospitals and the schools present insuperable difficulties to the preparation of a statement showing the actual cash value of the balance between contributions in money or kind made by the hospitals to the schools and by the schools to the hospitals; and this altogether apart from considerations as to the value of the work done by the schools for the hospitals. Frequently the ground on which the school buildings stand is the property of the hospital, but the buildings have heen erected by the school. In some cases parts of the buildings of the hospital are used conjointly for the purposes of hospital and school, especially in the case of pathological and bacteriological laboratories, and the apportionment of charges between two departments of the single institution would be a proper subject for the decision of experts. Another difficulty arises in connection with capital expenditure by the hospitals on account of the schools. In the case of St. Bartholomew's Hospital records of considerable debts, said to have been incurred by the school as far back as 1843, and still remaining undischarged, are extant; and it must be obvious that the yearly cost to the hospital of such debts, which appear to have borne no interest, could only be accurately estimated after an elaborate inquiry involving questions as to the constitution of the body it is sought to make liable, and the admission of any liability by such body, if it actually exists, whether legal or otherwise. The question of the value of land is also important and can only be satisfactorily dealt with by competent surveyors, as we find that the rateable value in the case of hospitals is an untrustworthy guide, even if the school buildings were capable of being separately rated, which in some instances is scarcely the case.

9. We have, therefore, thought it better to confine ourselves to statements of fact in regard to each hospital, and these will be found in the Third Appendix, which we adopt as part of our report. We believe the facts fairly cover the ground of inquiry, but the assesments of the money values which should be set against considerations which do not take the form of money payments must be left to professional valuers should it be thought desirable to further prosecute these inquiries.

10. We have, however, no hesitation in reporting that in the cases of King's College Hospital and University College Hospital, it may fairly be said that no money given or subscribed to those hospitals was, in the year 1903 (the last year for which the accounts are complete), contributed directly or indirectly by the hospitals for the maintenance of medical education.

11. In the cases of Guy's Hospital and the Royal Free Hospital, we doubt whether on consideration of all the circumstances (and especially in the case of Guy's Hospital to the facts stated by the Dean of the Medical School, see Appendix III.), the schools can be considered as deriving any pecuniary benefit from the hospitals.

12. In the case of all the other hospitals, namely: Charing Cross, the London, the Middlesex, St. Bartholomew's St. George's, St. Mary's, St. Thomas's, and the Westminster, we report that, in our judgment, contributions, either direct or indirect or both, were made in the year 1903, to the schools out of the funds of the hospitals.

13. With reference to the first part of the second head of inquiry—viz., whether any direct or indirect return for the contributions made by the hospitals to the schools is received by the hospitals from the medical schools—we have come to the following conclusions:—

14. (A) We find that, in some cases, the fact that a large body of students and of medical men are being, or have been, educated in a hospital, diffuses a wide interest in that institution and thus tends to aid the finances of the hospital.

15. (B) We find that the presence of a body of eager young men watching the proceedings of their teacher has the tendency to keep the medical man on the alert and to counteract the effects of the daily routine of duties; and that the opportunity for teaching a large number of pupils attracts to hospitals with schools the gratuitous services of the most eminent men in the profession.

16. (C) We think that the publicity which attends the work of a hospital where there is a body of young men in attendance, also tends to maintain at a high level the whole work of the institution.

17. (D) It has been urged before us that the great amount of work done without payment, or with inadequate payment, by students, in the character of medical clerks and dressers, and in connection with the out-patients and the casualty cases, constitutes a pecuniary advantage received by the hospital from the school; but the evidence satisfies us that the expenses incurred in hospitals with schools are generally in excess of those in hospitals without schools, and we are of opinion that no saving of expense can be attributed to the presence of medical students. On the contrary, some of the evidence before us, together with the study of the accounts of the various hospitals, has brought to our attention remarkable variations in the expenses incurred by the several hospitals, and raises the important question whether, in the case of some of the hospitals to which schools are attached, there is not considerable extravagance and waste in the expenditure.

18. (E) With regard to the welfare of the patients, this depends so largely on the character of the individual medical men and nurses concerned with each case that it is difficult to draw any line between the two classes of hospitals. Probably, in cases of great obscurity and difficulty, the presence of a large number of students may at times be useful; but on the other hand we think that the quiet of a hospital without students must often be a comfort to patients, and on the whole we do not think that the hospital, with schools can substantiate any superiority, in this respects over other hospitals.

19. (F) As regards the advancement of medical science and the consequent benefit to the public, the existence of a medical school is in our opinion of the highest value. London probably offers the greatest facilities of any city in the world for clinical teaching and for surgical and medical research, and we regard it as of the highest importance that the greatest use should be made of these facilities In making this statement, however, we feel bound to call attention to what we have said in a subsequent paragraph of our report (paragraph 25).

20. We cannot doubt that the presence of a medical school has a tendency to give to the management of a hospital a progressive character, as well as an interest in experiments tending to the advancement of medical and surgical science. But it seems to follow, as a consequence in such cases, that the real administration of the hospital will be placed mainly under the control of the medical staff, and that the expenditure of the hospital will thereby tend to increase. On the other hand, though the absence of a school may have a tendency to give to the management of the hospital a stationary character, and to leave the administration mainly in the hands of a lay body of managers, the evidence before us shows that some hospitals without schools are excellently conducted and can compare favourably with some hospitals with schools.

21. By the second part of the second head of reference we are asked to ascertain in each case, whether the direct and indirect return received by the hospital from its medical school is equivalent to the amount of the contribution made by the hospital to the school. Inasmuch as the returns, whether direct or indirect, are of a general kind and cannot be reduced to definite figures in money value, we find it impossible to give a categorical reply to this inquiry. But the general conclusion at which we have arrived is the following:—The schools confer certain considerable benefits on the hospitals, and the hospitals, by opening their doors to the students, provide a clinical laboratory and allow the students of the school to work there; this confers on them a very great benefit, because without the admission to the hospital the students could obtain little or no clinical teaching These mutual benefits may, we think, be fairly set off the one against the other.

22. If that be done, as we think it fairly may be, it follows that, in the case of the schools which last year received benefits in money or money's worth from the hospitals over and above the benefits last alluded to, there is no return made by the schools to the hospitals which can be treated as recouping this expenditure of the hospitals, and that (subject always to the observation made in paragraph 7) the schools still remain debtors to the hospitals in respect of the pecuniary contributions made to them

23. We answer the third specific head of inquiry by saying that, beyond the matters to which we have already referred, we do not find that any special considerations have been advanced in justification of the expenditure by the hospitals on the schools, or any general considerations which would apply to all hospitals having medical schools.

24. Such are our replies to the specific matters referred to us; but we desire to add that, in the course of our inquiries with a view to these replies, certain matters have come before us so closely connected with the subjects of the reference that we think it desirable to lay before your Royal Highness the impressions produced on our minds.

25. We have formed the opinion that a broad line of distinction ought to be drawn between the studies of the first three years of a medical student's curriculum and the studies of the last two years—or, in other words, between the preliminary and intermediate studies on the one hand and the final studies on the other; and that whilst the latter studies can only be pursued with advantage within the walls of a hospital, and nowhere in the world with more advantage than in London, the earlier studies have no real relation with a hospital, and are therefore more properly to be pursued in an institution of a university character; and further, that the attempt of many of the hospitals to associate with themselves schools teaching the preliminary and intermediate subjects is a great, if not the chief, source of the exhausted condition of the funds of many of the schools and the consequent demand of the schools on the funds of the hospital.

26. It is therefore with great satisfaction that we find that the Statutes of the University of London (paragraph 80) direct the Senate to "use its best endeavours whenever practicable to secure such common courses of instruction for Internal Medical Students in the preliminary and intermediate portion of their studies under Appointed or Recognised Teachers at one or more centres"; and if this object can be carried into effect we believe that it will free some of the schools from burdens which they find it difficult to bear.

27. The great uncertainty which, in many or all cases, attends any attempt to ascertain the intentions of the original founders of the hospitals and medical schools, the impossibility of knowing what were, from time to time, the exact motives in the minds of the subsequent donors and subscribers, and the complexity of the relations which have existed between hospitals and schools, seem to render any attempt to open up the transactions in the past between the hospitals and schools very undesirable, even if it were possible. But for the future the matter is different; and we venture to submit that the distinction between the hospital and the school should in every case be drawn, not only definitely and exactly, but with such clearness that it may be understood by the general public, and so that no question may arise as to the destination and application of moneys contributed, whether by the King's Fund or from any other source.

28. Finally, we cannot conclude this Report without thanking our Honorary Secretary for the great aid which he has afforded us throughout the course of our inquiries.

We have the honour to be, Sir,

Your Royal Highness's most obedient Servants,

Edw. Fry,

C. G. Stepney,

Welby.

J. Danvers Power, Hon. Secretary, February 8th, 1905.

Appendices containing minutes of evidence and particulars relating to the expenditure of 12 London hospitals on medical schools, laboratories, museums, libraries, are included in the volume, which is published at 2s. by Mr. George Barber, of 23 Furnival Street, Holborn, E.C.

